# What is the potential for social networks and support to enhance future telehealth interventions for people with a diagnosis of schizophrenia: a critical interpretive synthesis

**DOI:** 10.1186/1471-244X-13-279

**Published:** 2013-11-01

**Authors:** Gavin Daker-White, Anne Rogers

**Affiliations:** 1 Collaboration for Leadership in Applied Health Research and Care (CLAHRC) for Greater Manchester, The University of Manchester, 5th Floor, Williamson Building, Oxford Road, Manchester M13 9PL, UK; 2 Faculty of Health Sciences, Organisation and Delivery of Health Care Research Group, University of Southampton, Highfield, Southampton SO17 1BJ, UK

**Keywords:** Schizophrenia, Social networks, Peer support, Self-management, Telemedicine, E-health, Review, Meta-synthesis

## Abstract

**Background:**

Digital technologies are increasingly directed at improved monitoring, management and treatment of mental health. However, their potential contribution to social networks and self-management support for people diagnosed with a serious mental illness has rarely been considered. This review and meta-synthesis aimed to examine the processes of engagement and perceived relevance and appropriateness of telehealth interventions for people with a diagnosis of schizophrenia. The review addresses three key questions. How is the use of digital communications technologies framed in the professional psychiatric literature? How might the recognised benefits of telehealth translate to people with a diagnosis of schizophrenia? What is the user perspective concerning Internet information and communication technologies?

**Methods:**

A critical interpretive synthesis (CIS) of published findings from quantitative and qualitative studies of telehealth interventions for people with a diagnosis of schizophrenia.

**Results:**

Most studies were of an exploratory nature. The professional discourse about the use of different technologies was overlain by concerns with surveillance and control, focusing on the Internet as a potential site of risk and danger. The critical synthesis of findings showed that the key focus of the available studies was on the delivery of existing traditional approaches (e.g. improving medications adherence, provision of medical information about the condition, symptom monitoring and cognitive behavioural therapy). Even though it was clear that the Internet has considerable potential in terms of accessing and utilising lay support, the potential of communication technologies in mobilising of resources for personal self-management or peer support was a relatively absent or hidden a focus of the available studies.

**Conclusions:**

Based on an interpretive synthesis of available studies, people with a diagnosis of schizophrenia or psychosis use the Internet primarily for the purposes of disclosure and information gathering. Empowerment, regulation and surveillance emerged as the key dimensions of engagement (or not) with telehealth interventions. The findings suggest that telehealth interventions are disproportionately used by particular patient groups (e.g.women, people who are employed). Further research needs to ascertain the mechanisms by which telehealth interventions may be potentially beneficial or harmful for engagement and management to people with a diagnosis of schizophrenia.

## Background

The benefits of e-health are strategically important and include opportunities for social support and access to sources of information. People with a diagnosis of schizophrenia often face stigma, marginalization and associated social isolation [1-4]. There is an increased desire of marginalized patient groups to use the internet [5]. From a user perspective, the Internet has been seen to offer support from online initiatives such as the “e-patient movement” and as a complement or supplement to offline 'real world’ initiatives of self-management support [6]. However, the processes and outcomes of social networking activities for support and illness self-management in online contexts remain unclear. Including a network perspective is seen to offer an opportunity to address the broader set of (social) assets and resources available to people in need of illness management and support [7]. This article presents a synthesis of published studies concerning telehealth interventions aimed at people with a diagnosis of schizophrenia. The focus of the review is on studies involving participants with a diagnosis of schizophrenia. However, we also included “psychosis” as a search term to identify potentially relevant papers.^a^ For brevity, in the remainder of this article the term “people with a diagnosis of schizophrenia” is used, but with the aforementioned caveat.

Patients’ use of mobile technologies to enable improved clinical and self-management is increasingly becoming a key focus in health policy and practice. There has been significant research and development in furtherance of the objective to support a collaborative approach to managed self-care for long term conditions [8,9]. However, within a limited evidence base, questions remain concerning clinical and cost effectiveness. Initiatives for users of services with a diagnosis of psychosis are seen as potentially transformative, providing alternatives to conventional mental health services [5]. Key issues here concern the perceived acceptability or utility of the interventions (particularly in open systems) and factors in users’ engagement (or not) with new technologies. In a study of the potential benefits and harms of an Internet-based peer support network for people with depression, it was found that there was the potential for a “downward depressive spiral triggered by aggravated psychological burden” [10]. Internet chat rooms and messaging facilities have been used as part of a therapeutic community of people with personality disorders in a rural area of northern England, although the service has yet to be formally evaluated. An initial hesitancy to discuss problems online was related to concerns about burdening the system moderators, who were themselves service users [11].

Over the last three decades, services for people with a diagnosis of schizophrenia have increasingly been organised around the provision of care in open rather than closed settings, with less of a focus on hospitalisation. Recently, there has been a growing recognition of the potential for the transmission of health information and opportunities for social networking through digital health services. Increasing confidence is also placed on the potential power to transform communication, clinical practice and relationships. In relation to the Internet, online relationships have been found to offer immediacy and constancy, and unlike offline relationships, are seemingly not to the same extent bound by geographical, temporal or spatial constraints [6]. It is also a setting which has fed into user empowerment in mental health as it allows for the identification and use of experiential information [12]. However the nature, benefits and drawbacks of e-health for people diagnosed with serious mental health problems have not been studied extensively. The engagement of users is essential for developing extended possibilities for management and support in future interventions involving information and communication technology (ICT). It is also clear that it is important in the mental health field to explore the nature of the experience of the condition in relation to specific settings to understand the nuances of how information and support interventions are engaged with [13].

From an offline clinical perspective, the reasons for deficits in the social support networks of people with a diagnosis of schizophrenia centre on the behavioural characteristics of the patients themselves [14]. One purported thesis is that people with a diagnosis of schizophrenia experience a “social network crisis” at onset of symptoms [15] (although a longitudinal study found that gender, economic status and activity in the labour market were more important predictors of social network diversity [16]). One study found that individuals with a diagnosis of schizophrenia with greater 'social skills’ had larger social networks, although interestingly participants did not feel that they received greater support than did people with smaller social networks or more negative symptoms of schizophrenia [17]. The effectiveness of offline peer support groups was tested in a randomised, controlled trial which found that whilst they may improve the social networks of people with psychosis, these positive effects “did not generalize to other relationships; for instance, contact with family and friends” [18].

In this paper, we explore these issues through a meta-synthesis of literature on telemedicine and e-health for people with a diagnosis of schizophrenia. The review started with three key questions. How is the use of digital communications technologies framed in the professional psychiatric literature? How do the generally recognised benefits of telehealth translate to people with a diagnosis of schizophrenia? What is the user perspective concerning Internet information, self management and peer support? We set out to examine processes in telehealth interventions (e.g. engagement) and hoped to uncover findings in relation to user experiences and attitudes (e.g. perceived appropriateness). However, in Critical Interpretive Synthesis (CIS) it is not possible to know specifically what findings will be found in advance of searches and the aim is rather to interrogate a particular field of research [19].

## Methods

### Overview

The methods involved use of a pilot phase, study protocol, a broad and inclusive search strategy and the systematic appraisal, assessment and extraction of information from reports. We examined material that is not usually included in either qualitative or mixed-methods syntheses, including case reports. The extraction of findings, themes and concepts from key papers was completed independently by both authors. Several attempts were made to compare the findings of the studies against each other. This phase borrowed from the method of “translating” findings and concepts into one another as used in meta-ethnography [20]. This is a matrix-based approach that involves close comparison of findings and concepts as found in reports. It should be stressed that some 'qualitisation’ of statistical reports was involved here, as we were primarily interested in the ways in which the authors of quantitative studies conceptualised their findings and their implications. Initially, studies were examined critically according to methodological features including the nature of the settings, participants, interventions and outcomes. An elucidation of these processes and the methods by which a synthesis was constructed follows.

### Pilot phase

We set out to conduct an interpretive synthesis in order to construct a theoretical model of telehealth interventions for people with a diagnosis of schizophrenia. Pilot searches revealed a manageable number of studies, although they were mainly exploratory in nature, methodologically diverse and described a variety of interventions and technological platforms. For these reasons, an early decision was made to conduct a mixed-methods synthesis of the broad field. Critical Interpretive Synthesis [19] offers one of the few recognised ways of conceptually bringing together the findings of studies employing diverse designs and was adopted accordingly.

### Theoretical framework

This review attempted a wide definition of telehealth interventions, the specific limits of which can be seen in the search strategy (Table 1). The main focus was towards interventions that involved the use of telephones, networked computers, handheld devices (including mobile phones) and any experimental 'stand alone’ monitoring or communication devices. The main aim was to conduct a critical synthesis of the concepts and findings in research reports. Current theoretical strands concerning social networks, self-management and digital technology were employed as an interpretive lens. Critical Interpretive Synthesis (CIS) is designed to *critically interrogate the literature* of a particular area [19]. It is an iterative and cyclical process focused around the selected research interest in which research questions are used as a *compass* during the CIS process. The aim of CIS is to try to generate theory and or concepts in relation to a defined field of research.

**Table 1:** Search strategy – research studies on telehealth interventions in schizophrenia

	
1	(telehealth OR tele-health OR telemedicine OR tele-medicine OR internet† OR computer* OR web OR interactive OR telecommunication OR telephone OR phone OR SMS OR tele-monitor OR telemonitor OR telemanagement OR tele-management OR teleconsultation OR telecare OR tele-care OR telematic OR telepharmacy OR tele-pharmacy)
2	(psychosis OR schizophrenia)
3	1 and 2

*Not included in EMBASE (5905 results if retained), MEDLINE and PSYCHINFO as too many results were returned.

†Not included in MEDLINE, PSYCHINFO and Web of Knowledge (Social Sciences) as too many results were returned.

### Discourse analysis of professional literature

Traditionally, a 'digital divide’ has been widely reported in relation to the Internet, with for example older people and those from disadvantaged groups less likely to have access. On this basis, one would expect people with a diagnosis of schizophrenia to have lower rates of access, as they tend to be poorer or otherwise disadvantaged in relation to the general population. These issues were evident in the results of a UK population survey, where older respondents and those with lower levels of educational attainment were less likely to use the Internet than others. However, 18% of those surveyed had nevertheless used the Internet to find information about mental health [21]. More recently, whilst the availability of Internet access via mobile technologies such as smart phones has in theory increased access for some groups, a recent Armenian-based study has shown that mobile users do not engage in as many online activities as computer users do, thus decreasing their access to some potential benefits [22].

The greater surveillance, risk and controversy over best practice characterising the mental health field more generally is also an important consideration [23]. Although how this translates where telehealth is involved, e.g. in relation to the process of engagement, has not been explored. Thus, we initially undertook an analysis of psychiatric discourse, derived from hand searches of editorials and correspondence in selected psychiatry journals, in order to identify this as a first stage in the synthesis. The journals hand searched were American Journal of Psychiatry, British Journal of Psychiatry and The Psychiatrist (formerly Psychiatric Bulletin).

### Search and inclusion criteria

The review of reports of research studies was built on simple searches of the following bibliographic databases: ASSIA, British Nursing Index, CINAHL, EMBASE, ERIC, OVID MEDLINE, PSYCHINFO, Sociological Abstracts and Web of Science (Table 1). The titles, abstracts and key words of the articles found were assessed for inclusion. We included reports of any research studies (including feasibility studies, case studies, qualitative studies, surveys, cohort studies and randomised controlled trials (RCTs)) published in English in peer reviewed journals between 2000 and 2011. Other reviews were included, although none were found during the searches (see discussion for references to two relevant reviews published since). Short reports and research reports submitted as correspondence were included. Studies were excluded if they were not focused specifically on participants of people with a known diagnosis of schizophrenia or schizoaffective or shizotypal disorder. Studies would be excluded where reporting of findings or methodological features rendered them “unacceptable” or “fatally flawed” according to an assessment of design features, study aims and outcomes (see below). At assessment and data extraction, the reference lists of included studies were searched for additional references.

### Quality appraisal and data extraction

A protocol was drafted before commencement of searches and a data extraction form was developed see (Additional file 1). Extraction and appraisal was done initially by the first author, with a sub-set of papers assessed by both authors to conceptually enhance the synthesis process. A previous study has shown that inter-rater reliability is poor in relation to critical appraisal of qualitative research [24]. However, because interpretation is a subjective enterprise, it is beneficial for concepts to be extracted by more than one worker. Thus, in five articles that were considered key for synthesis, findings were extracted independently by both authors. The quality of reported research methods was evaluated using a set of criteria used in CIS, to wit: “Are the aims and objectives clearly stated? Is the design clearly specified and appropriate? Do the researchers provide a clear account of the process through which findings were produced? Do the researchers display enough data to support their interpretations and conclusions? Is the method of analysis appropriate and adequately explicated? Quality – Excellent/Acceptable/Unacceptable? If 'Unacceptable,’ Why?” [19].

### The three stages of synthesis

Synthesis of the extracted material was undertaken as an iterative process involving 3 different stages. Firstly, the studies were compared with each other as a whole, principally in terms of the nature of the samples, any methodological weaknesses and the main results and findings. This phase was informed by interpretive approaches, including meta-ethnography [20] and qualitative evidence synthesis [25], although statistical findings were also retained and compared. Results and findings were initially grouped into “pros and cons of telehealth technologies” and “effects on mutual peer support.” Following the comparison of these data, technologies were then broken down into four different groups ('open’ or unrestricted Internet use, 'closed’ web sites requiring username and passwords to access, handheld devices – originally separated into mobile telephones and handheld computers – and 'smart’ medication dispensers that record the times of bottle openings) for the purposes of comparison.

An assessment of the findings from phase 1 suggested that they fell into three main areas: control/surveillance, engagement/empowerment or marginalization/alienation and social networking or relationships. Following a return to the data extraction forms, a re-reading of the original articles, and a re-visit to phase 1, the final schema for synthesising the findings was constructed around four themes: Surveillance and control, engagement, social networks/study sample and therapeutic effects (or not) (phase 2). A final 'synthetic product’ [19] (phase 3) was realised by reflecting the findings from the studies against the framework derived from the discourse analysis of editorials and correspondence.

## Results

### Professional discourse on the internet and telepsychiatry

The results of the discourse analysis of editorials and correspondence are summarised in (Additional file 2: Table S1). It is relevant to note an initial exploration of the ways in which psychiatric literature portrays the merits of the Internet, or otherwise reflects concerns and tensions more generally about the provision of treatment and or management to people with a diagnosis of schizophrenia. The most prevalent themes and issues appeared overlain with tensions between the provision of information, the management of risk, surveillance and control, and encroachment of traditional areas of psychiatric jurisdiction. On the other hand, the professional literature also pointed to some potential benefits of telehealth, notably as a means of increasing access to services.

### Search results and initial assessment

The results of the electronic bibliographic database searches are shown in Table 2. The titles (and where necessary, abstracts) of the 2088 records in Table 2 were assessed and 40 were judged to meet the inclusion criteria and were obtained for assessment. Having obtained the articles, 19/40 (47.5%) were found to fit the inclusion criteria and were assessed in full. A further 4 papers were excluded following full assessment. Six articles were found by back searching of reference lists, 2 of which were excluded following full assessment. A further short research report was found during the hand searches for the discourse analysis and was incorporated into the main review. The only reason for excluding papers up to this stage was that they did not meet the inclusion criteria outlined above. In total, 25 articles were assessed in full, with 6 being excluded following full assessment.

**Table 2:** Search results, by database

**Database**	**Fields searched/limits**	**Results returned**
British Nursing Index	All fields	10
ASSIA	All fields	92
CINAHL	Words in subject heading	352
EMBASE	Keyword mapped to subject heading	1176
ERIC	All fields	388
OVID MEDLINE	Keyword mapped to subject heading	430
PSYCHINFO	Keyword mapped to subject heading	594
Sociological Abstracts	All fields	52
Web of Science	Topic [Set limited to “Social Science”]	402
	Total	3496
	Following removal of duplicates	2088

### Quality appraisal and data extraction

In the five key papers assessed by both authors, all were considered 'acceptable,’ except one article where one felt the work was 'excellent.’ Following discussion, the reporting of research methods was also considered to be 'acceptable’ in the article.^b^ In the twenty-five articles assessed in full, the reporting of research methods was judged to be unacceptable in 6 (24.0%), although they were not excluded solely on these grounds. Following assessment, studies were excluded for focusing on staff rather than people with a diagnosis of schizophrenia, because the computer terminal being assessed was based in a resource centre or because they were solely concerned with the quality or readability of Internet-based materials. Finally, studies concerned with video-conferencing in diagnosis or clinical interviews were excluded at the latter stages as they did not fit with the focus of the review. The final dataset comprised thirteen articles (see Additional file 3: Table S2. Across the studies, it was possible to discern research samples disproportionately biased towards well-educated people, younger people, females, the employed and those in relationships when compared with controls (where any existed) or the population of people with a diagnosis of schizophrenia in general.

### Phase one synthesis - comparing the studies with one another

**Figure 1:**
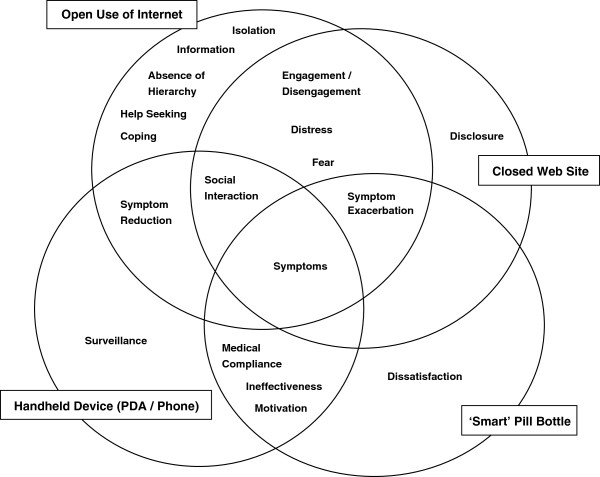
Themes found in research studies, according to technology type.

The phase one comparison of research findings according to technology type found that different themes appeared in the groups of research reports, but with some overlaps. These differences were found to be usefully presented as a Venn diagram (Figure 1). This diagram is a visual representation of one of the first attempts at representing the findings from the studies, stratified by technology group: open use of the internet, closed therapeutic web sites, smart medication bottles and handheld devices (including mobile phones and handheld computers). Each large circle in Figure 1 represents a different ICT technology. Within each circle are contained the themes that were found in studies using that device for intervention purposes. The diagram highlights that there are significant threats and potential disadvantages to all of these technologies for people with a diagnosis of schizophrenia. However, the most opportunities – or fewest threats – would seem to lie on the interface between open internet access and handheld devices.

### Phase two synthesis: therapeutic versus negative effects, by technology group

#### ***Open use of the Internet***

From Additional file 3: Table S2, the findings related to the open use of the Internet do not neatly translate into one another across the studies, but putting the concepts together it is possible to build a line of argument [20]. The articles included a case report [26], a content analysis of postings to Internet forums [27], a qualitative study of Internet use by people with a diagnosis of schizophrenia [28] and an RCT of Internet peer support [29]. The case study [26] and content analysis [27] of postings to schizophrenia discussion groups found open use of the Internet to be a beneficial way of increasing self-esteem and reducing symptoms. The qualitative study [28] is more useful as it was able to describe both the positives (relief, reassurance and reduction of fear) and negatives that may be associated with open use of the Internet. The latter were principally related to the psychological effects of being exposed to an uncontrollable quantity of potentially upsetting material that could exacerbate or provoke symptoms in the reader. The well educated and females were over represented in the study sample.

The RCT did not find positive outcomes [29]. However, the intervention was artificial in that the email list-server and the bulletin board used as interventions were devised by the research team and thus not 'naturally’ occurring Internet groups. Furthermore, given that the bulletin board required users to log in and the listserv depended on people providing and accessing email accounts, it is necessary to question whether this study was measuring 'open’ Internet access or not. Thus, this study might arguably be better placed alongside those concerned with “closed” web sites, although the study population was not a therapeutic group. Therefore, whilst it would be tempting to treat the findings of the RCT as being more trustworthy, it is questionable whether the same things are being 'measured’ as in the other studies of open Internet access. Online peer support may be conducted in public forums or in private ones requiring variable amounts of personal information from subscribing users. To date, the distinction between these different forms has been not been considered in the available studies. A salient theme of the RCT was that people who participated less in the groups showed less distress at follow-up than those who participated more frequently [29]. However, this finding was based on a *post hoc* analysis that involved putting some members of the intervention group with the control group for the purposes of the analysis.

The authors of the RCT commented that their findings related to the higher levels of distress seen in those who used the online groups more, or who reported more positive experiences of them were “counter-intuitive.” However, these findings fit with those from the survey showing that “excessive” Internet use was associated with an in increase in psychiatric symptoms. In this light, there is a need for future research to focus on issues around engagement or disengagement in online peer support, when disengagement might be seen as a marker of therapeutic success [30,31].

#### ***Closed therapeutic web sites***

The two articles comprised an RCT [32] and an exploratory, research and development report incorporating some results from focus group interviews with potential service users [33]. The latter was more useful for confirming other findings related to mobile phones and open Internet use (e.g. that computers can be frightening at certain stages of psychosis) [33]. The RCT showed how a therapist-moderated web site had led to a reduction in positive symptoms and an improvement in knowledge about schizophrenia [32]. The findings also confirmed those in studies of open Internet use that people with greater symptoms spent more time on the web site. However, the researchers rather postulated that in this 'closed’ intervention, the explanation offered was that users with higher rates of symptoms were seeking a bigger 'dose’ of the telehealth intervention [32].

#### ***Mobile phones and PDAs***

In contrast to the studies concerning Internet-based interventions, the studies in this group contained a higher proportion of male participants: 49 (79.0%) [34] and 34 (63.0%) [35] where figures were reported, although the case report involved an “intelligent” young female [36]. The only contribution of a focus group study (also of closed web sites) was to note that SMS-text messages could be “frightening” when the user was in a paranoid state [33]. Withdrawal and drop-out rates were high: 29% in the follow-up study [34] and 37.5% in a pilot for an RCT [37]. Thirteen per cent of participants to the feasibility and validity study of ecological momentary assessment were noncompliant [35]. Broadly, the findings from these studies were not encouraging with regard to SMS text messaging. The findings concerned with momentary ecological assessment via PDAs (handheld computers) were more encouraging, although they derived from a single case report and a feasibility and validity study. Thus, there is currently little evidence for the efficacy of these interventions and further research is indicated. However, a qualitative study that was published whilst this manuscript was in preparation has shown that smartphones can be successfully integrated into the everyday routines of those with a diagnosis of schizophrenia (see discussion, below).

#### ***Smart medication bottles to improve medications adherence***

As with the mobile phone/PDA studies, a higher proportion of the participants to these two studies were male: 95.5% in a pilot follow-up study [38] and 76.9% in an RCT [39]. In stark contrast to the other studies included in this review, 72.7% of the recruits to the pilot study were African Americans [38]. It was difficult to recruit patients to both studies, with a refusal rate of 40% in the case of the RCT [39]. In terms of medications adherence, the interventions appeared unsuccessful. However, the pilot study noted an improvement in knowledge about schizophrenia among caregivers and the RCT found fewer emergency and medical visits in the intervention group at 8 week follow-up [39].

### Phase three synthesis: surveillance and control—a continuation with off line relationships?

The material gathered for the discourse analysis that framed the synthesis (Additional file 2: Table S1) primarily pointed towards several meta-narratives that lay above and beyond the specifics of schizophrenia. Given that professional journals were hand searched, it was perhaps unremarkable that issues of regulation appeared as the predominant frame by which discussions on telepsychiatry and the Internet occurred. In professional accounts, the most frequently cited positives of the Internet related to increasing access and decreasing costs. The negatives were considerable and included the Internet as a portal to threats of disorder, misinformation and numerous dangers, as well as raising concerns around professional roles, boundaries, medico-legal or ethical issues and clinical power. At the same time, the discourse analysis – mainly via material incorporated as case studies – also pointed to the possible role of the Internet or World Wide Web as a foundational structure for delusions, thought broadcasting and other clinical features of the illness.

Reflecting the detailed findings of the discourse analysis against the research evidence, it was firstly apparent that attempts to increase medications adherence had been shown to be largely ineffective when the technology was at the more 'controlling’ end of the spectrum. This fits with the idea that patients in general have a tendency to resist the use of medications [40]. The use of technologies in this way was also seen (in clinical terms) to have increased paranoia and exacerbated symptoms in a sizeable minority of participants. More positive reports came from PDAs than from medication bottles. It should be stressed that prior to SMS (mobile telephones) and PDAs being collapsed into a single category for analysis purposes, the evidence suggested that PDAs were more user friendly than SMS-text based systems. However, as was noted above, the positive reports of a PDA derived disproportionately from a case report of a single, highly educated, young female patient.

It was noteworthy that whereas respondents to studies voiced antipathy towards 'automated’ SMS-text messages, similar reservations were seemingly not made with reference to PDAs. This may reflect issues of personalisation or the relationships that people have with their 'gadgets’ or handheld devices, although it is not possible to comment on these issues based on the findings in the available studies. However, the findings did point to a continuum of surveillance and control in telehealth technologies, ranging from open use of the Internet to 'smart’ medication bottles designed to increase medications adherence. This would suggest that some forms of technology may be better at engaging service users than others.

The open, “anonymous” and democratic nature of the World Wide Web that may act as doorway to threats and dangers simultaneously offers a very useful communication tool for people with “receptive and expressive interpersonal deficits” [41]. Looking across the research studies, however, this proposition does not fit with the finding that it was almost universally difficult to recruit to the studies and that drop-out rates were often high.

## Discussion

### Social networks and mechanisms for self-help or peer support

The main synthesis of findings and concepts appears in Table 3. The available evidence suggests that people with a diagnosis of schizophrenia use the Internet primarily as a forum for disclosure and as a means of gathering information about their symptoms and treatment. In the case of self-help, it is not always clear whether there are actually social networks in operation, or whether a more useful conceptualisation might simply consist of isolated individuals accessing information in order to help them in their daily lives and dealings with medicine. In a recent review of the role of social networks in chronic illness, a typology distinguished between dyadic relationships, affective communities and “networks of networks.” For people with a diagnosis of schizophrenia however, dyadic relationships and “affective communities” are problematic, as both involve a “level of expectations and pressure to conform” [42]. It is precisely these expectations and pressure that may make the 'anonymous’ Internet a more suitable place for people with a diagnosis of schizophrenia. The schema previously referred to also suggests that “networks of networks” are larger and more associated with better health outcomes than smaller ones [42]. This presents problems for people with a diagnosis of schizophrenia, as the (scant) findings above suggest a possible inverse relationship between the amount of time spent on-line and the number of real life friends. This would suggest that our current understanding of social networks in illness management needs to become multi-dimensional so that it may simultaneously incorporate 'real’ networks in geographical space, 'virtual’ networks in cyberspace as well as the inevitable intersect between the two. This finding also supports that found in an RCT of off-line peer support, where the intervention did not lead to social network benefits that went beyond the other study participants [18].

**Table 3:** Engagement, internet & peer support in schizophrenia

**Users’ perspective**	**Features of the internet**	**Clinical perspective**
Particular social groups (e.g. employed, educated females)	Internet as a social resource	Improves access to services
	[**ENGAGEMENT**]	
Self-esteem and self-validation	Internet as social leveller;	Erosion of professional roles
	Anonymity, absence of hierarchy	
	[**ENGAGEMENT**]	
Emotional and personal distance	Reduces Inter-personal Deficits	Management and Moderation
	[**ENGAGEMENT**]	
For help with daily problems	Information as social capital	Maintain professional power
	[**EMPOWERMENT**]	
Ambivalent needs for information	Uncontrollable amount of information	Internet as portal to misinformation and danger
	[**REGULATION**]	
Symptom exacerbation	A means of surveillance, monitoring and control	Information collected for clinical prerogatives
	[**SURVEILLANCE**]	

In social networking terms, the different technology groups covered by this review offer different opportunities. The 'smart’ medication bottles mainly serve the purpose of providing remote data to clinicians. To date, handheld devices have been used for similar purposes, although they could be used for peer-to-peer support, as proposed in a protocol for an exercise study [43]. In social networking terms, only Internet sites – whether 'open’ or 'closed’ – incorporating bulletin boards, forums or chat rooms can offer opportunities for social networking. However, given that the primary social capital of the Internet for people with a diagnosis of schizophrenia concerns information, it is a moot point whether or not people, say, reading each others’ illness blogs constitutes a form of active or ongoing social networking that might be harnessed for contact and support. Answers to such questions will require future research.

A qualitative study of mobile-phone based clinical assessment for psychosis recently published in this journal found that whilst the use of mobile and smart phones were “well integrated” into users’ everyday activities, “In some cases mobile-phone assessment led to a preoccupation with ones thoughts, and comparisons between the individual’s real, desired and past mental states.” Repetitiveness was further identified as a “likely barrier to long-term adoption” [44]. These results further point to the balance of benefit to the amount of time spent completing 'tasks’ related to ICT interventions and the possibility of over-use engendering detrimental preoccupations related to symptoms and mood state.

A potential benefit of the 'closed’ or moderated/controlled approach could be that it facilitates a form of close virtual “geodesic distance”[45] that might more easily transfer positive health or psychological benefits, including relief, reassurance and better coping strategies. Unfortunately, these outcomes were not considered in the RCT of a closed web site, which was solely concerned with symptoms and knowledge of schizophrenia. In terms of understanding how social networks can impact on illness management there is a need for an understanding of how 'distance’ operates in virtual networks and whether there are more useful understandings of social connections that go beyond simple notions of geographical distance.

There is growing recognition of the potential benefits of access to telehealth for mental health users [5] and of the work undertaken by users in relation to embedding and adapting to the potential of these interventions [46]. Exploring and utilising social networking to support the clinical and everyday self-management and activities of people has been recognised, but less so in relation to mental health. We explored these issues in this synthesis and found that the opportunities offered by the technologies need to be considered in light of factors relating to engagement, empowerment, regulation and surveillance. Particular groups of people with a diagnosis of schizophrenia come to the Internet for help with daily problems, self-esteem and self-validation. They did so because the Internet is a useful communication medium to engage mutual support whilst maintaining a degree of emotional and personal distance. The findings suggest that in line with considering the off line service response, diagnosis *per se* is relatively irrelevant. Rather, specifiable behavioural risks (to self and others) perceived by users and clinicians on the one hand, and patient centredness or empowerment on the other, are of most relevance in considering user engagement. The dominance given to technological issues and a pre-occupation with traditional clinical considerations suggests that the potential of telehealth in terms of social support, networking and utility to users has not to date featured significantly enough.

The supposed alienating and isolating features of the Internet make it a useful networking tool for people with a diagnosis of schizophrenia. Whilst it has been shown that the transmission of happiness along social networks is to a significant degree dependent on proximity [45], the available evidence might suggest that some people with a diagnosis of schizophrenia are not using the Internet for emotional reasons, with the notable exception of disclosure, although further research is clearly indicated here (see also below). If things get 'too emotional’ then symptom exacerbation may be the result. Thus, in this instance, emotional geodesic distance may be of therapeutic benefit when the aim is to access information about services, medications and one’s condition. The offline growth in the users’ movement may also allow for more online development of mutuality and support in relation to the monitoring, interpretation and management of mental health related problems. In these terms, the results of this synthesis around technological engagement match those found in a study of refugee women in the UK, where the outcomes of engagement included psychological empowerment and the development and maintenance of personal identity [47]. Thus, there may be a distinction (or differences in outcomes or side-effects) between interventions focused purely on access to information or peer networks, when compared with those focused on therapeutic or clinical intervention or monitoring.

A further tension exists between the stereotypical (or real) view that people with psychotic disorders may pursue a solitary existence, and the evidence across the literature that the groups who are accessing on-line services, contain proportionately more women, more formally well educated people, more likely to be employed and in relationships with others. This realisation begs questions about the nature of services, the social construction of increased accessibility, and the possible ratio of threats to benefits that telepsychiatric interventions might hold for different populations. More importantly, this further underlines the questions that remain about the relationship between offline and online social networks and wider assets and resources. For example, the biases in the samples of included studies point to ICT interventions as being disproportionately utilised by people who may already be richer than other people with a diagnosis of schizophrenia in terms of social assets and network connections.

### Limitations

The limits of the search strategy in different databases could mean that some relevant studies may have been missed. The incorporation of material from editorials and correspondence is novel; to our knowledge the first time such material has been brought into a systematized review. However, given that this may be contentious to some readers, this material was collected and analyzed separately from the main synthesis. Finally, although we were mainly concerned with the findings contained in the included studies, we have interpreted this material partly according to our own research interests in social networks and peer support. Given that most of the studies we found were not principally concerned with social networks or peer support, however, the findings of our efforts need to be considered principally as an agenda for future research. The added value of our approach when compared with other styles of review or synthesis is that we have been able to point to the relevance of themes for assessment in future studies, such as subjective perceptions of surveillance or control, the capacity for health-related information to cause harms as well as benefits and a need to disentangle the effects of technologies from those of interventions.

## Conclusions

A critical interpretive synthesis has offered useful insights into studies of telehealth interventions in schizophrenia which can inform further research in the field. Since the study described in this manuscript was completed, a Cochrane review of psycho-educational interventions utilising communication or information technology for people with a diagnosis of schizophrenia has been published [48]. Following the assessment of six trials with over 1,000 participants (published before October 2010) the reviewers found no significant differences in primary outcomes compared with standard care and concluded that ICT “has no clear effects compared with standard care, other methods of delivering psychoeducation and support, or both” [48]. A recent narrative review concluded that videoconferencing in patient assessment is “reliable and feasible,” and that promising initial success has been demonstrated in some aspects, especially telephone-based interventions aimed at improving medications adherence [49]. However, the reviewers did not apparently employ systematic means of appraising the reports or extraction of data and findings. Whereas both of these other reviews point to a weakness of evidence on efficacy, and a need for further research, our approach has also enabled us to point to factors or issues to be interrogated or investigated in future studies.

The findings of this synthesis underline that the Internet is a means of exchanging information or facilitating social contact, much like its use for other patients groups with a long term illness. Thus, the Internet is not only an intervention of itself, but is also the medium through which intervention may occur. The overall finding was that the Internet holds potential benefits and harms for people with a diagnosis of schizophrenia. Further research is indicated into the mechanisms of 'information overload’ [10] and its attendant psychological sequelae. It has been noted that the use of the Internet as a 'social skill’ is affected by existing structural inequalities and access to social resources. Thus, the “transformational potential . . . requires capacity building to overcome the effects of other, independent, structural sources of disadvantage” [50]. To date, telehealth interventions for people with a diagnosis of schizophrenia have focused on alternative means of delivering pre-existing services, such as therapy, attempting to increase adherence to medications and symptom monitoring. These approaches limit the socially transformational possibilities of digital communications technologies for people with a diagnosis of schizophrenia. In the light of this, the apparent failure of 'smart’ medications dispensers and other interventions focused on the continued surveillance, control and containment of people with a diagnosis of schizophrenia is perhaps unsurprising.

This review suggests that there are both similarities and differences between Internet use for those with a diagnosis of schizophrenia and those without. Over and above the delivery of a specific form of management, and like other review studies of Internet use [51], our review suggests that people with a diagnosis of schizophrenia use telehealth and the Internet in a variety of ways. This includes the searching for and use of information about medication, being supported and maintaining relationships with others, and learning from others about what it is like to manage their conditions. There was also some fear of the possible consequences of contact with others, with some unable or unprepared to deal with communication about negative or disturbing aspects of the condition via the Internet. The potential of social networking through online relationships offers the opportunity to enhance patient engagement and self-management as a compliment or alternative to traditional clinical responses. A personalised system to assist patients with a diagnosis of schizophrenia to self-manage and manage everyday life by monitoring symptoms and treatments remotely using mobile telecoms might in the future be usefully supported with additional online access to peer support from users and remote information. This review suggests that consideration needs to be given to the content, structure and management of such a facility.

## Endnotes

^a^ Some of the articles found referred to patients with a schizoaffective disorder or schizotypal (personality) disorder, although they were not excluded on this basis. Given the small number of studies, it was not possible to analyze results by sub-groups according to different diagnoses.

^b^ Although there was little difference in the findings extracted by both workers, AR (who has more experience of the mental health field) tended to add conceptual interpretations from the wider literature at an early stage, whereas GD-W was focused purely on the material contained in the reports at this stage.

## Competing interests

The authors declare that they have no competing interests.

## Authors’ contributions

GD-W participated in the design and execution of the study and drafted the manuscript. AR conceived of the study and participated in its design, execution and drafting of the manuscript. Both authors read and approved the final manuscript.

## Pre-publication history

The pre-publication history for this paper can be accessed here:

http://www.biomedcentral.com/1471-244X/13/279/prepub

## Supplementary Material

Additional file 1 Data Extraction form: Critical Interpretive Synthesis, Schizophrenia & Telemedicine. Click here for file

Additional file 2: Table S1 Main arguments and issues found in hand searched correspondence and editorials in three psychiatry journals. Click here for file

Additional file 3: Table S2 Main data extraction table. Click here for file
