# Supplementary material for: What is the potential for social networks and support to enhance future telehealth interventions for people with a diagnosis of schizophrenia: a critical interpretive synthesis
Source: BMC Psychiatry. 2013 Nov 1;13:279. doi: 10.1186/1471-244X-13-279 (PMC3917697; doi:10.1186/1471-244X-13-279)
Supplement: Additional file 1 — Data Extraction form: Critical Interpretive Synthesis, Schizophrenia & Telemedicine. [file 1471-244X-13-279-S1.doc]

**Additional file 1**: Data Extraction form: Critical Interpretive Synthesis, Schizophrenia & Telemedicine

| **Paper ID** |  | | |
| --- | --- | --- | --- |
| Date |  | | |
| Extracted by |  | | |
| Title of Article |  | | |
| Publication Date |  | | |
| Type of article |  | | |
| **Study details** | Location / Country |  | |
|  | Research question / Objectives |  | |
| **Discipline** | Or multi-disciplinary |  | |
| **Quality** | Are the aims and objectives clearly stated? | | Yes / No |
|  | Is the design clearly specified and appropriate? | | Yes / No |
|  | Do the researchers provide a clear account of the process through which findings were produced? | | Yes / No |
|  | Do the researchers display enough data to support their interpretations and conclusions? | | Yes / No |
|  | Is the method of analysis appropriate and adequately explicated? | | Yes / No |
|  | QUALITY? | Excellent / Acceptable / Unacceptable | |
|  | If ‘Unacceptable,’ Why? |  | |
| **Participants** | Population |  | |
|  | Age |  | |
|  | Age (mean/range) |  | |
|  | Gender (M/ F) |  | |
|  | Ethnicity |  | |
|  | Recruitment / sampling (inclusion criteria, response rates, diffs. between responders and non-responders) |  | |
| **Intervention** | (e.g. peer support, CBT) |  | |
| **Technology** | (e.g. web site, chat room, mobile device) |  | |
| **Social Networks** | Nature, size and type of social network considered |  | |
| **Outcomes** | What is being evaluated? |  | |
| **Data Collection** | Methods |  | |
|  | Validation (quantitative papers) or trustworthiness (qualitative papers) |  | |
| **Data Analysis** | Method |  | |
| Is it primarily descriptive? | |  | |
| Is it an ‘exploratory’ study, pilot or protocol? | |  | |
| How are results presented? | |  | |
| **Main findings**: Theme / Outcome #1 | |  | |
| **Main findings**: Theme / Outcome #2 | |  | |
| **Main findings**: Theme / Outcome #3 | |  | |
| **Main findings**: Theme / Outcome #4 | |  | |
| **Main findings**: Theme / Outcome #5 | |  | |
| **Main findings**: Theme / Outcome #6 | |  | |
| **Main findings**: Theme / Outcome #7 | |  | |
| **Main findings**: Theme / Outcome #8 | |  | |
| **Memos (i.e. implications for developing concepts and theories)** | |  | |
| **Meta-narratives (i.e. assumed concepts and theories)** | |  | |
| **Comments (Limitations, reviewer comments, etc.)** | |  | |
| **References** – Possible new | |  | |
| **References** – For Background | |  | |
