# Supplementary material for: What is the potential for social networks and support to enhance future telehealth interventions for people with a diagnosis of schizophrenia: a critical interpretive synthesis
Source: BMC Psychiatry. 2013 Nov 1;13:279. doi: 10.1186/1471-244X-13-279 (PMC3917697; doi:10.1186/1471-244X-13-279)
Supplement: Additional file 2: Table S1 — Main arguments and issues found in hand searched correspondence and editorials in three psychiatry journals. [file 1471-244X-13-279-S2.docx]

Additional file 2: Table S1 – Main arguments and issues found in hand searched correspondence and editorials in three psychiatry journals^[[1]](#endnote-1)^

| Discourse | Data |
| --- | --- |
| The Internet as a useful information resource | Major hallucinogenic drug information sites “quite accurate” [[2](#_ENREF_2)]  A source of help for people who self harm [[3](#_ENREF_3)]  Lots of people are looking for information about mental health problems on the Internet [[4](#_ENREF_4)]  “Accuracy of information appears to be more of a concern to commentators than actual users” [[5](#_ENREF_5)] |
| The Internet as danger with needs for regulation | E-suicide note found to be a hoax [[6](#_ENREF_6)]  Most medical web sites not considered “legitimate” [[7](#_ENREF_7)]  Patient got suicide drug via an “overseas pharmacy on the internet” [[8](#_ENREF_8)]  Misinformation, unhelpful or even harmful [[3](#_ENREF_3)]  Patients contemplating suicide should not surf the Internet [[9](#_ENREF_9)], [[10](#_ENREF_10)]  A misleading source of information [[11](#_ENREF_11)]  “The dissemination of bizarre beliefs on a much wider scale than ever before” [[11](#_ENREF_11)]  “Make possible and spread shared delusional ideation”[[11](#_ENREF_11)]  “Internet addiction appears to be a common disorder”[[12](#_ENREF_12)]  Sexual preoccupations [[12](#_ENREF_12)]  Doctors should be careful about what they reveal online [[13](#_ENREF_13)]  “Disastrous consequences such as identity fraud, harassment, stalking or worse, acts of violence” [[13](#_ENREF_13)]  Making personal information available could be harmful for the individual [[14](#_ENREF_14)]  “Fertile ground for subcultural discussions” [[5](#_ENREF_5)] |
| [ACCESS] Telepsychiatry is useful in rural locations | Suicide in rural young men [[15](#_ENREF_15)]  Suicide in the elderly [[16](#_ENREF_16)]  “Itinerant therapeutic community”[[17](#_ENREF_17)] |
| [ACCESS] Internet good for people at home | E-maternity leave [[18](#_ENREF_18)]  Elderly people do not like visiting medical centres [[16](#_ENREF_16)]  A solution for people with anxiety or phobias [[19](#_ENREF_19)] |
| [ACCESS] Internet as a conduit to mainstream services | “Stepped care”[[20](#_ENREF_20)]  Intended to operate “outside treatment time” [[17](#_ENREF_17)]  Internet-therapies benefited from weekly telephone prompts [[21](#_ENREF_21)] |
| Professional erosion, roles, boundaries | Preponderance of “public forums”[[22](#_ENREF_22)]  Self-help with professional interaction [[23](#_ENREF_23)]  Medico-legal issues [[23](#_ENREF_23)]  Resistant professionals with concerns [[24](#_ENREF_24)]  “In the end, it is easier to program and reprogram computer[s] ... than to train and retrain clinicians”[[20](#_ENREF_20)]  “Extending clinician’s reach”[[20](#_ENREF_20)]  The need for direct human contact [[25](#_ENREF_25)] *contra* [[26](#_ENREF_26)][Also in [[27](#_ENREF_27)]]  “Worryingly, the idea that ECT is barbaric is all over the Internet”[[28](#_ENREF_28)]  The Internet search revealed that the boy’s parents had been murdered [[29](#_ENREF_29)]  “The meaning of the patient’s texting ultimately needs to be understood through expressive therapy” [[30](#_ENREF_30)]  Research on internet addiction is based on “inconsistent criteria” [[31](#_ENREF_31)]  The relationship between suicide and the internet has not been properly researched [[31](#_ENREF_31)]  “The term ‘internet addiction’ is a caricature of a diagnosis rather than a well-explored entity”[[32](#_ENREF_32)]  “Blogs may change the doctor-patient relationship” [[14](#_ENREF_14)] |
| The Internet as a means of surveillance and control | Internet delusions [[33](#_ENREF_33)]  Perception broadcast / Becoming a camera [[34](#_ENREF_34)]  Audits, outcomes, surveillance [[23](#_ENREF_23)]  Clients remain anonymous [[35](#_ENREF_35)]  ‘Googling’ about patients for clinical purposes [[36](#_ENREF_36)]  “Perfect execution of computer code modelling evidence-based clinical decision making” [[20](#_ENREF_20)]  Cf: “The challenge that some online communities represent to traditional diagnostic criteria” [[11](#_ENREF_11)]  “Containment of a high level of disturbance”[[17](#_ENREF_17)]  “The use of blogs as a source of clinical material raises ethical and legal issues”[[14](#_ENREF_14)] |
| Use of resources | A ‘disconnection’ between patients and health-care providers [[16](#_ENREF_16)]  Online services are cheaper [[24](#_ENREF_24)]  But only for large numbers of patients [[23](#_ENREF_23)]  Save time and costs [[37](#_ENREF_37)]  The labour-intensive nature of psychotherapy [[20](#_ENREF_20)]  The demand exceeds the resources [[26](#_ENREF_26)]  “Leaner, greener, digital public services”[[5](#_ENREF_5)] |
| Social interaction, disclosure | Online relationship with another boy with a seizure disorder [[38](#_ENREF_38)]  Easier via computer interface [[23](#_ENREF_23)]  “Only 53% were agreeable to being contacted by text message”[[39](#_ENREF_39)]  Improves social support, well-being, social networking [[14](#_ENREF_14)]  “The need to self-disclose ... through a blog would decrease when a person feels ‘contained’ by mental health professionals.”[[14](#_ENREF_14)]  Disclosure in Internet versus face-to-face interactions [[27](#_ENREF_27)]  Valued anonymity, more likely to disclose [[27](#_ENREF_27)]  “Anonymity, privacy and convenience are valued” [[5](#_ENREF_5)] |

1. Parker I: *Discourse dynamics: Critical analysis for social and individual psychology*. Florence, KY, USA: Frances/Routledge; 1992.

2. Bogenschutz MP: **Drugs on the internet**. *Am J Psychiatry* 2001, **158**(12):2094-2095.

3. Prasad V, Owens D: **Using the internet as a source of self-help for people who self-harm**. *Psychiatric Bulletin* 2001, **25**(6):222-225.

4. Cummings EJ: **Trends in mental health googling**. *The Psychiatrist* 2009, **33**:437.

5. Powell J: **Young people, self-harm and internet forums: Commentary on . . . Online discussion forums for young people who self-harm**. *The Psychiatrist* 2011, **35**:368-370.

6. Ball D: **A virtual cry for help?** *The British Journal of Psychiatry* 2000, **177**:568.

7. Charatan F: **American psychotherapy goes on the internet**. *BMJ* 2000.

8. Beatson S: **Suicide and the internet**. *The Psychiatrist* 2000, **24**:434.

9. Thompson S: **Suicide and the internet**. *The Psychiatrist* 2001, **25**:400.

10. Prior TI: **Suicide methods from the internet**. *Am J Psychiatry* 2004, **161**(8):1500-1501.

11. Vila-Rodriguez F, Macewan BG: **Delusional parasitosis facilitated by web-based dissemination**. *Am J Psychiatry* 2008, **165**(12):1612.

12. Block JJ: **Issues for DSM-V: internet addiction**. *Am J Psychiatry* 2008, **165**(3):306-307.

13. Raffi A, Mahmoo J: **Internet and doctors' security - how exposed are you?** *The Psychiatrist* 2009, **33**:438.

14. Wuyts P, Broome M, McGuire P: **Assessing the mental state through a blog: psychiatry in the 21st century?** *The Psychiatrist* 2011, **35**(10):361-363.

15. Henderson S: **Focus on psychiatry in Australia**. *Br J Psychiatry* 2000, **176**:97-101.

16. Jones B: **Suicide among the elderly: the promise of telecommunications**. *British Journal of Psychiatry* 2002, **181**:191-192.

17. Rigby M, Ashman D: **Service innovation: a virtual informal network of care to support a 'lean' therapeutic community in a new rural personality disorder service**. *The Psychiatrist* 2008, **32**:64-67.

18. Bremer J: **E-maternity leave**. *Am J Psychiatry* 2001, **158**(7):1160.

19. Bai YM, Lin CC, Chen JY, Liu WC: **Virtual psychiatric clinics**. *Am J Psychiatry* 2001, **158**(7):1160-1161.

20. Greist J: **A promising debut for computerized therapies**. *Am J Psychiatry* 2008, **165**(7):793-795.

21. Andrews G: **Utility of computerised cognitive-behavioural therapy for depression**. *Br J Psychiatry* 2010, **196**(4):257-258.

22. Halpern J, Pope H: **Drs. Halpern and Pope Reply**. *Am J Psychiatry* 2001, **158**:2095.

23. Marks I: **Psychiatry in the future: Information technology can pull mental health care into the 21st century**. *The Psychiatrist* 2004, **28**:319-320.

24. Jones R, Leonard S, Birmingham L: **Setting up a telepsychiatry service**. *The Psychiatrist* 2006, **30**:464-467.

25. Markowitz JC: **How "supportive" is internet-based supportive psychotherapy?** *Am J Psychiatry* 2008, **165**(4):534; author reply 534-535.

26. Litz B: **Dr. Litz Replies [correspondence]**. *Am J Psychiatry* 2008, **165**(4):534-535.

27. Jones R, Sharkey S, Ford T, Emmens T, Hewis E, Smithson J, Sheaves B, Owens C: **Online discussion forums for young people who self-harm: user views**. *The Psychiatrist* 2011, **35**:364-368.

28. Joshi N: **Internet electroconvulsive therapy**. *The Psychiatrist* 2009, **33**:155.

29. White H: **Locating clinical boundaries in the World Wide Web**. *Am J Psychiatry* 2009, **166**(5):620-621.

30. Neimark G: **Patients and text messaging: a boundary issue**. *Am J Psychiatry* 2009, **166**(11):1298-1299.

31. Bell V: **Taking an internet history**. *Br J Psychiatry* 2009, **194**(6):561-562; author reply 562.

32. Cooney G, Morris J: **Authors' reply**. *Br J Psychiatry* 2009, **194**:562.

33. Compton MT: **Internet delusions**. *South Med J* 2003, **96**(1):61-63.

34. Schmid-Siegel B, Stompe T, Ortwein-Swoboda G: **Being a Webcam**. *Psychopathology* 2004, **37**:84-85.

35. Postel MG, de Jong CA, de Haan HA: **Does e-therapy for problem drinking reach hidden populations?** *Am J Psychiatry* 2005, **162**(12):2393.

36. Neimark G, Hurford MO, DiGiacomo J: **The internet as collateral informant**. *Am J Psychiatry* 2006, **163**(10):1842.

37. Stankard P, Younus S: **Forensic telepsychiatry**. *The Psychiatrist* 2007, **31**:155.

38. Daley ML, Becker DF, Flaherty LT, Harper G, King RA, Lester P, Milosavljevic N, Onesti SJ, Rappaport N, Schwab-Stone M: **Case study: the internet as a developmental tool in an adolescent boy with psychosis**. *J Am Acad Child Adolesc Psychiatry* 2005, **44**(2):187-190.

39. Donaldson A, Tayar Z: **Mobile telephone text messaging of clinic appointments in psychiatry**. *The Psychiatrist* 2009, **33**:39.

1. This discourse was captured by hand searching the editorials and correspondence published in three professional psychiatric journals. Any articles concerned with the world wide web or telemedicine were included and these searched were not schizophrenia-specific. Parker’s criteria for distinguishing discourses were adopted, including that a discourse has ideological effects; reproduces power relations; supports institutions; and, “reflects on its own way of speaking” [1]. The journals American Journal of Psychiatry, British Journal of Psychiatry and The Psychiatrist (formerly Psychiatric Bulletin) were hand searched from 2000 to 2011 inclusive. Correspondence, editorials and other non-research papers were searched for pieces about telemedicine or the World Wide Web. The hand searches were supplemented by electronic searches of these journals using the keywords ‘telemedicine’ and ‘telepsychiatry.’ [↑](#endnote-ref-1)
