# Supplementary material for: What is the potential for social networks and support to enhance future telehealth interventions for people with a diagnosis of schizophrenia: a critical interpretive synthesis
Source: BMC Psychiatry. 2013 Nov 1;13:279. doi: 10.1186/1471-244X-13-279 (PMC3917697; doi:10.1186/1471-244X-13-279)
Supplement: Additional file 3: Table S2 — Main data extraction table. [file 1471-244X-13-279-S3.docx]

Additional file 3: Table S2: Main Data Extraction Table

|  |  |  |  |  |  |
| --- | --- | --- | --- | --- | --- |
| Title | Technology Group / Method | Surveillance & Control | Engagement | Social Networks / Relationships | The potential of the technology |
| Internet forums: a self-help approach for individuals with schizophrenia? [[1](#_ENREF_1)] | Open use of the Internet  Content Analysis | “No informed consent was obtained as the postings were publicly available without restriction. The authors did not participate in the groups, so that the group processes were not influenced” (Methods, p.475). | “Emotional interaction e.g. empathy or gratitude were comparatively rare” (Abstract, p.474)  “Discussions about daily problems of the illness like symptoms, medication and emotional involvement with the illness ... [were] central topics” (p.475) | “Self-help mechanisms mostly used are disclosure and providing information” (Abstract, p.474) | “A useful approach to cope with alienation and isolation” (Abstract, p.474) |
| How patients with schizophrenia use the Internet: qualitative study [[2](#_ENREF_2)] | Open use of the Internet  Qualitative Study | “Internet information was described as leading to a more critical attitude towards one’s own medication” (p.7)  “Information from the Internet had the potential to significantly change the relationship with the attending doctors” (p.7)  “Depended on the quality of the patient-doctor relationship” (p.7)  “Participants reported that they rarely spoke to their doctors about the results of their Internet searches, partly due to the fear that the doctors might feel criticised” (p.8) | “Participants were often conflicted between interest in the topic and the fear of finding out more potential side effects” (p.5)  “to help them find a suitable facility or to better evaluate services before deciding to use them” (p.6)  “the advantage of not being confronted with insecurities in personal contact” (p.7)  “the special content of the information gathered in this way” (p.7)  Ambivalence “regarding the need for information” (p.9)  “It became evident that patients with schizophrenia may perceive only a certain amount of information as reasonable and feel the need to guard themselves against excess information.” (p.9) | Anonymity and absence of hierarchy of on the web (p.6)  “a lower perceived threshold to accessing information and . . . confidence for overcoming problems with social interaction” (p.6)  “Self-help or mutual help in coping with the illness, boosting self-esteem and self-validation through sharing one’s story” (p.7)  “A specific advantage for patients with psychosis, who often have pronounced fears and uncertainties in social interaction, was not having to face another person but still being able to gain information and interact with others without feeling devalued or unsafe.” (p.9)  “A large proportion of our sample was well educated” (p.9) | Relief, telling own story, reassurance, reduce fear (p.7)  “Better coping strategies or lower thresholds for seeking help” (p.7)  “Stimulus overflow . . . inability to deal with the abundance of information, problems with concentration, lack of energy and depressive symptoms, paranoid ideas and fear of symptom provocation and the wish to distance oneself from illness-related topics as part of the recovery process.” (p.6)  “sadness, and hopelessness . . . in relation to dramatic illness stories” (p.7) |
| Internet peer support for individuals with psychiatric disabilities: a randomized controlled trial [[3](#_ENREF_3)] | Open use of the Internet  RCT |  | “Some kind of facilitation . . . may be important to produce positive effects” (p.60) | 2/3 women recruited via web sites and e-news lists.  Engaged in services, well educated, employed, c.50% married.  “Baseline levels of social support were higher than expected.” + higher QoL scores (p.60)  “Control group members were more likely to have been homeless” (p.60)  “Some individuals reported a lack of connection to other group members. Perhaps, the unmoderated, unstructured format may have affected the groups’ ability to achieve a sufficiently strong sense of community, a vital component of peer support.” (p.60) | “Counterintuitive findings demonstrating those who report more positive experiences also experienced higher levels of distress” (Abstract, p.54)  “Positive outcomes were not found” (p.60)  “Individuals in the low participation group reported less distress at 12 months than at baseline” (p.60)  “In the high participation group, distress increased significantly from 4 months to 12 months” (p.60)  “The evidence-base remains insufficient for drawing any firm conclusions about the value of Internet peer support groups, and caution should be taken before restricting access or discouraging their use.” (p.61) |
| Case study: the Internet as a developmental tool in an adolescent boy with psychosis [[4](#_ENREF_4)] | Open use of the Internet  Case Study |  | “Mark felt very comfortable in his virtual world, which allowed increased reality testing within the framework of supportive therapy” (p.189) | “Mark used a chat group for children who have seizure disorders. This experience allowed him to share his worries and thoughts with other youngsters with seizures. He established a close friendship with John, a boy with a similar problem. These contacts helped decrease Mark’s sense of isolation. He was able to have online relationships without the stress of face-to-face interactions” (p.188)  “From social isolation to integrating back into school with his peers” (p.189)  “The Internet allowed Mark to socialize with peers and assisted him in his home study” (p.188) | Internet as “interactive play therapy” (p.190)  “Mark’s family reported increases in his level of confidence and self-esteem as he progressed in these complex games.” (p.188)  “As a teenager he started making his own web sites and experienced relative freedom from psychotic symptoms as he entered cyberspace. In particular, Mark described being able to ignore ‘the voices’ or actually push them out of his mind while he was engaged in computer-based activities. Mark’s parents specifically reported that he was not observed talking to himself while online.” (p.188) |
| Elevated social Internet use and schizotypal personality disorder in adolescents [[5](#_ENREF_5)] | Open use of the Internet  Survey |  | “It is plausible that individuals with SPD would be drawn to the Internet because it is a venue in which receptive and expressive interpersonal deficits are less likely to reciprocate in exclusionary behaviour from peers.” (Introduction, p.53) | “The number of ‘real life’ interpersonal friendships was not associated with time spent emailing, but was significantly negatively correlated with time spent in a chat-room, and showed a strong negative trend for time spent participating in on-line games.” (p.55)  “Thus adolescents with fewer friendships spend more time in chat-rooms on the Internet” (p.55)  Those in the ‘No disorder’ and ‘Other personality’ disorder groups showed “higher mean frequency of friendships than the SPD group, p<0.05),” baseline measurement (p.54)  “On the Internet, individuals with SPD may not be immediately excluded due to abnormal nonverbal behaviour as is likely to occur in real-life interactions” (p.55) | “Research indicates that excessive use [of the Internet] is associated with psychiatric symptoms” (Abstract, p.50)  “Greater Internet use may be a consequence of the symptoms or a contributor to symptoms” (p.56) |
| Web-based psycho-educational intervention for persons with schizophrenia and their supporters: one-year outcomes [[6](#_ENREF_6)] | Closed web site  RCT | “Each forum was led and moderated by a therapist” (p.1100) | “Cumulative web site usage and symptom severity indicated that individuals with more severe positive symptoms tended to spend more time on the SOAR site (r=.65, p=.005) and to access SOAR more frequently (r=.62, p=.009)” (p.1103)  “The visual display of words on a monitor may help compensate for deficits in auditory processing, attention, and memory and may improve concentration and attention to the task” (pp.1103-4, discussion) | The sample were mostly female and educated | “Counterparts in the telehealth group had significant and large reductions in positive symptoms during the treatment intervention (t=-2.06, df=97, p=0.42, d=-0.88).” (p.1102)  “In the telehealth group a significant and large improvement in knowledge about the diagnosis of schizophrenia (t=-2.34, df=24, p=0.028, d=0.88) (p. 1102)  “Higher web site usage was associated with higher rates of positive symptoms, suggesting that those most in need of treatment sought and used a bigger ‘dose’ of the telehealth intervention” (p.1103) |
| Supporting young people with psychosis in the community: an ICT enabled relapse prevention tool – [UN-PUBLISHED PIECE] [[7](#_ENREF_7)] | Closed web sites & Mobile-phones  Mainly focus group data used herein | “Management of the system by an active case-worker was considered important” (p.vi) | “little enthusiasm for SMS based systems, partly because of cost (they’re always out of phone credit), and especially if the messages they receive are automated rather than of human origin” (p.vi)  “It is preferred that the style and timing of access be chosen by the client” (p.vi)  “ Little enthusiasm for ‘motivational messages’ (p.vi)  “Concerns about using ICT in certain frames of mind or at certain stages of psychosis or recovery – during intense psychosis computers can be difficult or frightening .” (p.vi)  “users would be motivated to use an online intervention . . . with the ability to connect with their friends” (p.vii) | “intelligent technologies which combine social networking and web-based treatment to promote independent home based care” (Abstract, p.i)  “The client group envisaged a web-based system with three functions: 1. Asynchronous, ongoing communication with centres staff . . . 2. Client-to-client social networking . . . 3. While many said they did not enjoy CBT, they recognised the value of it and felt it should be part of the intervention.” (pp.vi-vii)  “The system needs to bring users into the world, not allow them to hide within an institution or organisation-based social network” (p.viii)  “Social networking is important and Facebook is significant in clients’ lives. Clients felt that some of the best advice they had received during clinical programs came from fellow clients.” (p.vi) | “When in a paranoid state SMS could be frightening” (p.vi) |
| The efficacy of SMS text messages to compensate for the effects of cognitive impairments in schizo-phrenia [[8](#_ENREF_8)] | Mobile phones  Follow-up / Outcomes study | Only patients who staff felt had “impaired goal-directed behaviour” were invited to take part.  “After the intervention medication adherence dropped significantly” – having only been 8% higher during the intervention (p.269) | 18 (29%) dropped out during the study.  “It is worth noting that medication use does not reach sufficient mean frequency to be fully effective even during the intervention.” (p.271) |  | “Keeping appointments with mental health workers and carrying out leisure activities increased with prompting, while medication adherence and attendance at training sessions remained unchanged” (Abstract, p.260)  “Prompting with SMS text messages” led “to an increase in success percentage” (p.267)  “The overall mean success percentage over all goal categories was 47% (across patients SD 27.9) during baseline, increased to 62% (SD 20.1) during the intervention, and dropped to 40% (SD 31.7) at follow-up” (p.267)  “The mean success percentage actually decreased somewhat with prompting in the non-responder group [NS]” (p.269)  “Subjective evaluation . . . 19 patients (41%) thought the SMS text messages to be effective, 15 patients (33%) were neutral towards the efficacy of the SMS text messages, and 12 patients (26%) evaluated the SMS text messages as ineffective” (p.270)  “A considerable number of goals were still not achieved.” (p.271) |
| Mobile interventions for severe mental illness: design and preliminary data from three approaches [[9](#_ENREF_9)] | Mobile phones  Pilots for RCT  (Sub-study about bipolar disorder excluded) | Intervention 1: Experience sampling via SMS text messaging “to facilitate case management”  Intervention 2: Group functional skills training including between session mobile phone contacts with therapists | Of 8 participants, 3 withdrew, 2 “had very prominent negative symptoms and had difficulty maintaining motivation to continuously engage in the text exchanges and in responding to the text messages in the allotted time.” (p.719)  “none of the participants dropped out of the intervention” (p.720) | Liked receiving calls weekly to help practice skills (p.720) | “25% showed a substantial drop in responsiveness in the second half of the trial.” (p.719)  “Greater improvement in functional outcomes” than controls (p.720) |
| Use of Palm computer as an adjunct to cognitive-behavioural therapy with an ultra-high-risk patient: a case report [[10](#_ENREF_10)] | Real-time collection of data on thoughts, moods and behaviour  Case Report |  | “It is unclear whether the use of Palm computers would work in UHR patients who have more average intelligence” (p.7) | “Her experience of loneliness remained unchanged” (p.5)  “During the assessment in month 3, Andrea’s activities consisted primarily of ‘surfing’ the Internet or ‘doing nothing.’ However, over the course of treatment, her activities appeared to become more diversified. At the same time, she continued to spend the great majority of her time at home alone in her room.” (p.5) | “The use of the Palm computer was acceptable to the patient and resulted in a substantial increase in homework completion. This methodology resulted in rich information about the patients’ daily functioning and patterns of improvement during treatment. The experience sampling method data were also successfully used in the application of treatment interventions.” (Abstract, p.1)  “Andrea reported reductions in her experience of anxiety, guilt and depression/sadness.” (p.5)  “as treatment progressed, Andrea reported fewer difficulties expressing her thoughts, as well as increased ability to recognize dysfunctional thoughts. Additionally, she reported being more interested in her own activities with a greater sense of her own competency. She also appeared to be more hopeful about her future.” (p.5)  “Her BDI-II score decreased from 37 (admission) to 17 (month 8). . . . her SIPS / SOPS ratings of positive symptoms also declined from 17 to 8 between admission and month 5 (month 8 ratings were unavailable). Similarly, her negative symptoms and disorganized symptoms declined from 22 to 14 and from 9 to 4 repectively.” (p.5) |
| Feasibility and validity of computer-ized ecological momentary assessment in schizophrenia [[11](#_ENREF_11)] | Real-time collection of data on thoughts, moods and behaviour | “It may provide important information that is inaccessible via standard clinical and functional outcome measures administered in the laboratory” (Abstract, p.507)  Noncompliant “individuals demonstrated greater overall cognitive impairment (M Global Impairment T-score = 25.54, SD=7.19) than compliant participants (. . . T-score 34.22, SD 7.49), t(46)=-2.67, P<0.05).” (pp.509-510)  “From a clinical point of view, EMAc may also permit treatment advances through verification of medication compliance and the completion of desired exercises or the detection of early warning signs of relapse.” (p.513) | “Seven participants (13%) were noncompliant with the EMAc procedures (demonstrating no more than 4 completed assessments or the equivalent of one full day of participation)” (p.509) |  | “EMAc was experienced positively by the sample as a whole. Most notably, the average ratings by participants indicated few difficulties with the EMAc methodology and a high willingness to participate in similar studies in the future.” (pp. 510-511)  “Concerning adaptive behaviours, participants with higher ILSS total scores were more likely to be at work or school (γ_01_ = 4.739, P<.05), visiting the homes of friends or family (γ_01_= 4.025, P<.01), shopping or doing chores outside the house (γ_01_= 3.739, P<.05), and having interactions with strangers and people other than family or friends (γ_01_= 4.280, P<.05)” (p.511) |
| Feasibility of using the MED-eMonitor system in the treatment of schizophrenia: a pilot study [[12](#_ENREF_12)] | Smart Pill Bottle  Pilot follow-up study | “To enhance medication and research protocol compliance” (Abstract, p.283)  “The patient’s personal and occupational functioning” (p.283)  “Ease of central evaluation by treating professionals” (Abstract, p.283)  “A third of the patients endorsing they felt increased paranoia as a result of using the monitor, and with one patient believing the monitor made his symptoms worse.” (p.286) | Noted that it was “difficult” to recruit patients and caregivers (p.289)  “It is possible that patients who were so impaired that they could not give consent also would have been too impaired to use the device” (p.288)  22 patients were recruited but only 14 actually used the device at home. The study was completed by 17 patients and 12 caregivers.  21 male and 1 female were recruited. 72.7% were African Americans  6 v. 4 caregivers “reported that they would not like to continue using the monitor after the study” (p.286) |  | “there was significant improvement in knowledge of schizophrenia for caregivers (t=-2.39; p=0.048)” (p.286)  “Most of the patients stated that the monitor was easy to use and they took their medication more regularly as a result. More than half [9 versus 7 patients] indicated that they would like to continue using the monitor after the study is over” (p.286)  3 “experienced symptom exacerbations during the study period; 1 experienced a symptom exacerbation home but prior to beginning to use it; 3 discontinued using it during the study period for no stated reason; and 1 took the monitor home but never used it, again for no stated reason.” (p.286) |
| Tele-monitoring of medication adherence in patients with schizophrenia [[13](#_ENREF_13)] | Smart Pill Bottle  RCT | “Early warnings about impending nonadherence” (Abstract, p.675)  “Patients participating in studies are more willing than others to adhere to treatment protocols” (p.682)  “Even when patients knew they were being monitored, they still had drug free days and apparently increased their tablet consumption prior to appointments” (discussion, p.682)  “Future plans . . . may need to incorporate psycho-educational sessions . . . to enhance uptake of the platform” (p.682) | “The rate of study refusal was 40%” (p.682)  “Most patients (69.4%, n=25) were able to use the interface unaided after one or two demonstrations each lasting for about 1 hour. Five (13.8%) patients required assistance throughout the study and the remaining 6 (16.6%) required four to five demonstrations each lasting for about 1 hour.” (p.681) |  | “Fewer medical and emergency visits in [smart pill bottle group ] (p=0.01 and p=0.001) at study end point [8 weeks post randomization]” (p.681) |

References

1. Haker H, Lauber C, Rossler W: **Internet forums: a self-help approach for individuals with scizophrenia?** *Acta Psychiatr Scand* 2005, **112**:474-477.

2. Schrank B, Sibitz I, Unger A, Amering M: **How patients with schizophrenia use the internet: qualitative study**. *J Med Internet Res* 2010, **12**(5):e70.

3. Kaplan K, Salzer MS, Solomon P, Brusilovskiy E, Cousounis P: **Internet peer support for individuals with psychiatric disabilities: A randomized controlled trial**. *Soc Sci Med* 2011, **72**(1):54-62.

4. Daley M, Becker D, Flaherty L, Harper G, King R, Lester P, Milosaljevic N, Onesti S, Rappaport N, Scwab-Stone M: **Case Study: The Internet as a Developmental Tool in an Adolescent Boy With Psychosis** *Journal of the American Academy of Child & Adolescent Psychiatry* 2005, **44**(2):187-190.

5. Mittal VA, Tessner KD, Walker EF: **Elevated social Internet use and schizotypal personality disorder in adolescents**. *Schizophrenia Research* 2007, **94**(1-3):50-57.

6. Rotondi AJ, Anderson CM, Haas GL, Eack SM, Spring MB, Ganguli R, Newhill C, Rosenstock J: **Web-Based Psychoeducational Intervention for Persons With Schizophrenia and Their Supporters: One-Year Outcomes**. *Psychiatric Services* 2010, **61**(11):1099-1105.

7. Lederman R, Wadley G, Gleeson J, Alvarez-Jimenez M, Spiteri-Staines A: **Supporting Young People With Psychosis in The Community: An ICT Enabled Relapse Prevention Tool**. *PACIS 2011 Proceedings* 2011.

8. Pijnenborg GHM, Withaar FK, Brouwer WH, Timmerman ME, van den Bosch RJ, Evans JJ: **The efficacy of SMS text messages to compensate for the effects of cognitive impairments in schizophrenia**. *British Journal of Clinical Psychology* 2010, **49**:259-274.

9. Depp CA, Mausbach B, Granholm E, Cardenas V, Ben-Zeev D, Patterson TL, Lebowitz BD, Jeste DV: **Mobile interventions for severe mental illness: design and preliminary data from three approaches**. *J Nerv Ment Dis* 2010, **198**(10):715-721.

10. Kimhy D, Corcoran C: **Use of Palm computer as an adjunct to cognitive-behavioural therapy with an ultra-high-risk patient: a case report**. *Early Interv Psychiatry* 2008, **2**(4):234-241.

11. Granholm E, Loh C, Swendsen J: **Feasibility and validity of computerized ecological momentary assessment in schizophrenia**. *Schizophr Bull* 2008, **34**(3):507-514.

12. Ruskin PE, Van Der Wende J, Clark CR, Fenton J, Deveau J, Thapar R, Prasad M, Kehr BA: **Feasibility of using the Med-eMonitor system in the treatment of schizophrenia: A pilot study**. *Drug Information Journal* 2003, **37**(3):283-291.

13. Frangou S, Sachpazidis I, Stassinakis A, Sakas G: **Telemonitoring of Medication Adherence in Patients with Schizophrenia**. *Telemedicine and e-Health* 2005, **11**(6):675-683.
